# Functionalized and Nonfunctionalized Nanosystems for Mitochondrial Drug Delivery with Metallic Nanoparticles

**DOI:** 10.3390/molecules28124701

**Published:** 2023-06-12

**Authors:** Shashi Kiran Misra, Jessica M. Rosenholm, Kamla Pathak

**Affiliations:** 1 School of Pharmaceutical Sciences, CSJM University Kanpur, Kanpur 208024, India; shashisarthak@gmail.com; 2 Pharmaceutical Sciences Laboratory, Faculty of Science and Engineering, Åbo Akademi University, BioCity (3rd Floor), Tykistökatu, 6A, 20520 Turku, Finland; 3 Faculty of Pharmacy, Uttar Pradesh University of Medical Sciences, Saifai, Etawah 206130, India

**Keywords:** metallic nanoparticles, mitochondrial dysfunction, antioxidants, reactive oxygen species, calcium homeostasis, biocompatibility

## Abstract

**Background:** The application of metallic nanoparticles as a novel therapeutic tool has significant potential to facilitate the treatment and diagnosis of mitochondria-based disorders. Recently, subcellular mitochondria have been trialed to cure pathologies that depend on their dysfunction. Nanoparticles made from metals and their oxides (including gold, iron, silver, platinum, zinc oxide, and titanium dioxide) have unique modi operandi that can competently rectify mitochondrial disorders. **Materials:** This review presents insight into the recent research reports on exposure to a myriad of metallic nanoparticles that can alter the dynamic ultrastructure of mitochondria (via altering metabolic homeostasis), as well as pause ATP production, and trigger oxidative stress. The facts and figures have been compiled from more than a hundred PubMed, Web of Science, and Scopus indexed articles that describe the essential functions of mitochondria for the management of human diseases. **Result:** Nanoengineered metals and their oxide nanoparticles are targeted at the mitochondrial architecture that partakes in the management of a myriad of health issues, including different cancers. These nanosystems not only act as antioxidants but are also fabricated for the delivery of chemotherapeutic agents. However, the biocompatibility, safety, and efficacy of using metal nanoparticles is contested among researchers, which will be discussed further in this review.

## 1. Introduction

Mitochondria, one of the vital cell organelles, is notable as being oval or bean shaped, as well as for containing a dynamic branched system that consistently divides and fuses in accordance with the regulation of fission and fusion theory [1]. Mitochondria are the energy generator of eukaryotic cells, in addition to producing the adenosine triphosphate (ATP) that performs diverse functions, including the biosynthesis and degradation of protein molecules, cell division and respiration, as well as controlling membrane potential. They are adorned with countless cristae in the inner membrane of mitochondria, which are specifically involved in the generation of ATP on a massive scale. Besides the generation of ATP, mitochondria also perform numerous other tasks, such as the biogenesis of amino acids, ROS signaling, calcium ion homeostasis, apoptosis, stem cell monitoring, and the control of innate immunity [2]. Further, the bidirectional drive between the nucleus and ATP generator is tightly regulated via the fission (inner membrane) and fusion (outer membrane) of mitochondria. Diverse physical platforms (including signaling pathways, protein–protein interaction, and the regulation of both calcium homeostasis and released reactive oxygen species) occur in the powerhouse of the cell. A number of metabolic processes, such as glycolysis, the Krebs cycle, oxidative phosphorylation, and acetyl CoA oxidation, are executed in the mitochondria, both for the purpose of ATP generation, as well as for dumping released electrons from the various pathways.

The mitochondria has an extremely flexible ultrastructure, intended for the regulation of the bioenergetics flux of the cell. The literature maintains that the sequence of mitochondrial genome (MtDNA) can stabilize maternally inherited diseases. Additionally, the proteomics of mitochondria has described approximately 1000 proteins inside the nuclear genome, of which a mere 13 proteins are coded by the mitochondrial DNA. Hence, quality control of mitochondria is critically important to avoid genome defects, as well as to maintain cell metabolic homeostasis [3]. The production, regulation, and consumption of organic molecules are highly required for supporting cell growth and proliferation. Mitochondrial oxygen utilization is key process that generates ATP from these organic compounds and other valuable intermediates. Besides ATP generation, reactive oxygen species (ROS) are also produced during the process of oxidative phosphorylation. These ROS mediate the signaling pathways associated with several basal and adaptive responses controlling both cell and organism hemostasis [4].

A myriad of mitochondrial targeted approaches were set up for targeting the mitochondrial proteins and signaling pathways concerned with the biogenesis and dynamics of mitochondria. The enhancement of antioxidant bioavailability via the supply of natural products is a fascinating strategy to address mitochondrial dysfunction. Nanoengineered novel drug delivery systems offer advanced tools and techniques for successfully delivering active drugs to the targeted site (mitochondria). In this context, several polymeric, vesicular, and metallic nanoparticles have been effectively developed for the alleviation of mitochondrial defects.

## 2. Mitochondrial Dysfunction and Diseases

**Figure 1:**
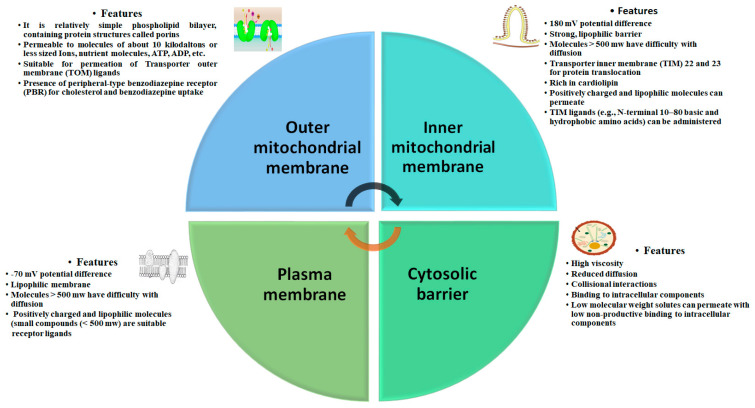
Interconnected distinguishing features and processes occurring in the mitochondria and associated sites.

Human cells comprise more than a thousand replicas of mitochondrial DNA (MtDNA). At the time of birth, these are homoplasmy that may be altered due to pathogens or mutation, and can consequently develop wild type MtDNA, or heteroplasm. The threshold of pathogenic MtDNA varies from person to person, and from organ to organ in the same individual. Abnormal mitochondria dynamics (i.e., fission and fusion), reduced oxidative phosphorylation capacity, and a transformed electron transport chain can each elicit ATP scarcity, alter ROS with poor membrane potential, and cause specific stress signaling responses. The resultant poor mitochondrial protein transportation influences enzyme assembly, which initiates several consequences, such as oxidative damage, proteotoxic stress, mutation, and the exhaustion of MtDNA. The altered morphology and functions of mitochondria can interrupt their nutrient requirements, substrate availability, and genetic signals, causing inherited MtDNA mutations which thus weaken its defense system. Figure 1 outlines the prominent features of the outer, inner, associated plasma membrane and cytosolic barriers.

**Figure 2:**
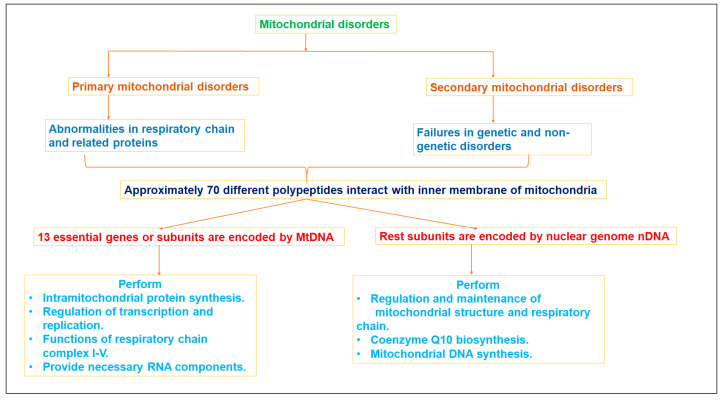
Mitochondrial disorders (primary and secondary) and their operational issues.

Clinical studies have revealed that progressive aging is implicated with mitochondrial impairment, and that the release and accumulation of ROS plays a crucial role in pathogenesis of age-related issues, including neurodegenerative disorders and diabetes. The imbalanced level of generation and oxidation of ROS disturbs the respiratory chain function of mitochondria, altering not only outer/inner membrane permeability, but also calcium homeostasis and high heteroplasmic MtDNA in the sufferers [5]. Mitochondrial abnormality (either by MtDNA or nDNA) may occur at any age, and can affect single or multiple organs, e.g., the eye and ear. Both Leber hereditary optic neuropathy and nonsyndromic hearing loss (or deafness) have been reported as being due to abnormal mitochondrial functions. The literature reports noticeable clinical features, such as pigmentary retinopathy, cardiomyopathy, diabetes mellitus, and disorders in central nervous system (i.e., ataxia, dementia, seizures, fluctuating encephalopathy, and migraine), as being associated with the abnormal functioning of mitochondria. Figure 2 illustrates primary and secondary mitochondrial disorders and their operational issues.

Recently, Friedreich’s ataxia (a neurodegenerative defect) has been identified due to mitochondrial imbalance of ROS and reactive nitrogen species (RNS). One of the mitochondrial proteins, named frataxin, is encoded and arranged by the FXN gene, which is tremendously expressed in tissues with high metabolic rates. Frataxin is vitally involved in accumulation of the iron–sulfur clusters required for energy production. The latest evidence proposes that this protein deactivates the glutathione peroxidase enzyme, elevates thiol levels, and detoxifies the ROS associated with Friedreich’s ataxia. Hence, targeting this protein may be a possible approach for a drug delivery system [6]. Genetic disorders such as Wilson’s disease, hereditary spastic paraplegia, and ethylmalonic aciduria are caused by the mutation of ATP7b, SPG7, and ETHE1 genes, respectively [7]. Table 1 compiles different diseases caused by damaged mitochondria, as well as the primary methods of their management. 

## 3. Therapeutic Approaches

Approaches such as ‘one size fits all’ and ‘precision medicine’ are often employed for the mitigation of primary mitochondrial disorders. The former manages symptomatic interventions (based on diet, exercise, and pharmacological therapeutics) that initiate mitochondrial biogenesis, provoke nitric acid synthetase, amplify ATP synthesis, control mitochondrial autophagy, and stimulate fission/fusion processes. On the other hand, the precision medicine approach focuses on specialized therapies, i.e., nucleotide supplementation, swapping of damaged MtDNA, gene replacement remedies, the elimination of noxious metabolites, and organ transplantation, if required. Table 2 compiles several therapeutic agents for the regulation of the myriad pathways involved in impaired mitochondria.

These approaches may represent a common tactic for the management of primary mitochondrial disorders and can improve an individual’s quality of life, but they are unable to eradicate the cause of the mitochondrial disorder completely.

## 4. Biological Barrier and Toxicity

There are myriad barriers to designing mitochondria-targeted drug delivery, such as biological barriers and drug toxicity. After reaching to the target cell, intracellular diffusion across the outer and inner mitochondrial membrane is another challenge. Further, mitochondrial complex (I–IV) inhibition within ETC from drugs such as haloperidol and thiothixene have shown critical toxicities [26]. Resultantly, neurological disorder such as Parkinson’s disease arise. Nanotechnology-based approaches are promising tools for effectively targeting mitochondria, owing to their narrow size and their efficiency in transporting bioactive agents. Both the mitochondrial membranes experience variable permeability, i.e., the outer membrane allows the permeation of a large array of small molecules, whereas the inner membrane is quite selective for transportation. The reason behind selective permeability through the inner mitochondrial membrane is the presence of specialized channel proteins there [27]. Further, the transition pore of the outer mitochondrial membrane is wider than the inner mitochondrial membrane, which facilitates the faster traversal of therapeutics. The highly folded inner mitochondrial membrane is hard and possesses narrow transition slits to separate out the mitochondrial matrix within the inter membrane space. This characteristic morphology of mitochondria also poses a barrier for the passage of drug molecules across matrix. The aforesaid issues create hurdles for targeting mitochondria-based disorders. Several aspects, including high negative membrane potential (−160 to −180 mV), unambiguous protein import mechanism, variable lipophilicity, and idiosyncratic phospholipid composition of inner membrane, are a few key factors considered while designing a mitochondrion-targeted drug delivery system [28]. Permeation of the outer membrane of mitochondria is concentration dependent, following passive diffusion. A negative surface charge and mitochondrial membrane potentially drive the production of ATP, in addition to exploiting for mitochondrial targeting through cationic ions and drug molecules via electrostatic interaction. Cardiolipin, another exclusive phospholipid-based component, is confined and produced in the inner mitochondria region. It plays a central role in processing several reactions and processes required for mitochondrial dynamics and respiratory chain complexes [29]. Moreover, electrochemical composition and membrane potential are remarkably distinct, compared to cytoplasm. A mitochondrion comprises its own genome in form of a circular DNA containing 37 genes and 16,500 base pairs, essential for protein synthesis. The synthesized 13 proteins are basic components of the mitochondrial electron transport chain, as well as performing the process of oxidative phosphorylation [30]. Mitohormesis is related to the damaged adaptive response of mitochondria, and was very first discussed by Ristow et al. in 2010. This process controls mitochondrial homoeostasis and assists organismal senescence. The literature supports that increased age favors defects in mitochondria (mitophagy), replacing new mitochondria via the process of fission and biogenesis [31]. Therefore, the mitochondria’s basic functions deteriorate with aging, and can bring about several age-related disorders. In this context, drug-induced cell and mitochondrial toxicities are extensively demarcated in the geriatric population into myriad categories of pharmacological agents [32]. For example, troglitazone (an antidiabetic drug) exhibited mitochondrial toxicity and was withdrawn from the market after liver dysfunction was reported. The drug potently disrupted mitochondrial-oriented respiration by inhibiting complexes II, III, IV, and V [33]. Another, nefazodone (a serotonin antagonist) has been withdrawn from the market in United States in the year 2004 due to profoundly inhibiting mitochondrial respiratory chain in isolated rat liver cells [34]. Biguanides (buformin, metformin, and phenformin) trigger lactic acidosis, which is directly associated with mitochondrial damage due to a surge in lactate production and respiratory inhibition [35]. An over-the-counter drug, aspirin is the most prescribed medicine in elderly patients for overcoming of pain and has been reported to inhibit respiratory processes, as well as opening MPT pores and disturbing of glutathione status in mitochondria [36]. Another cholesterol lowering drug, simvastatin, obstructs the ETC complexes I, IV, and V, augments calcium release, reduces mitochondrial membrane potential, and thus decreases ATP levels [37]. Further, both the in vivo administration of impoverished pharmacokinetic properties and the erratic biodistribution of drugs create challenges for the design of delivery systems. In many instances, therapeutic agents lost their potency with the addition of selective coding for the targeting of mitochondria.

## 5. Nanoengineered Mitochondria Targeted Approaches

Nanotechnology is able to advance the pharmacokinetic and biodistribution profiles of various drug molecules without altering their pristine molecular form. The nanoformulation of drugs can modify the inherent physicochemical attributes of drugs, such as solubility, lipophilicity, half-life, and minimization of immunogenicity. Successful delivery of bioactive substances with the use of nanoparticles can be realized by customizing particle size, lipophilicity, surface charge, and the addition of specific targeting moieties. Being biocompatible and biodegradable, nanoparticles have proven potential for site-specific delivery [38], in addition to the intracellular targeting of subcellular compartments. It is evident that the architecture of mitochondria is highly distinct from other subcellular parts in eukaryotic cells. The occurrence of proton pumps in the inner mitochondrial membrane creates positive charges on the inner folded surface, whereas a negative charge is found in the mitochondrial matrix. This charge gradient develops noticeable transmembrane potential across both outer and inner mitochondrial membranes [39]. Molecules such as pyruvates and lipophilic cations (i.e., triphenylphosphonium, rhodamine123, and tetrachlorotetraethylimidacarbocyanine) have been reported on for targeting mitochondria. The cationic lipoidal content of these molecules enables their permeation into the mitochondrial matrix, thus effectively targeting the region of interest. Positively charged nanoparticles induce an electrostatic interaction with the anionic phospholipids of mitochondrial membranes, and are then internalized. Inside the membrane, the nanoparticles rupture and release their drug with the mitochondrial matrix [40].

Recently, the cationic liposome-like vesicles known as ‘DQAsomes’ have been highly researched as mitochondria-targeted carriers, being explored to deliver cytotoxic therapeutics or DNA into the highly negative environment of cancer cells [41], as these cells experience hyperpolarized membrane potential (−220 mV) compared to the mitochondrial membrane potential of normal cells (−140 mV). Bolalipids containing DQAsomes are vesicle-like structures that are tuned for the delivery of DNA, genes, and peptides inside the mitochondrial matrix. The entrapped component is selectively transported via adorned protein pump machinery on the surface of mitochondria. DQAplexes are hybrid assemblies, comprising membrane liposomes with plasmid DNA for delivery of chemotherapeutics (specifically, into the mitochondria). Vaidya et al. conjugated folic acid on the surface of paclitaxel-entrapped DQAsomes to target overexpressive folate receptors on HeLa (tumor) cells. Outcomes obtained from confocal laser scanning microscopy revealed the enhanced antitumor activity of the designed functionalized DQAsomes, compared to bare DQAsomes [42]. However, the modi operandi for restricting the precise mitochondrial targeting of this system were unclear. Other nanoengineered dendrimers with high generation number are abundantly promoted to deliver cytotoxic agents to the mitochondria. Cations (such as triphenylphosphonium, polyamidoamine, and rhodamine) containing dendrimers encompass high net-positive charges and had the ability to carry genetic materials and chemotherapeutic agents at the cell organelle [43]. Table 3 summarizes the various nanodrug delivery systems utilized for the management of damaged mitochondria-based diseases.

**Figure 3:**
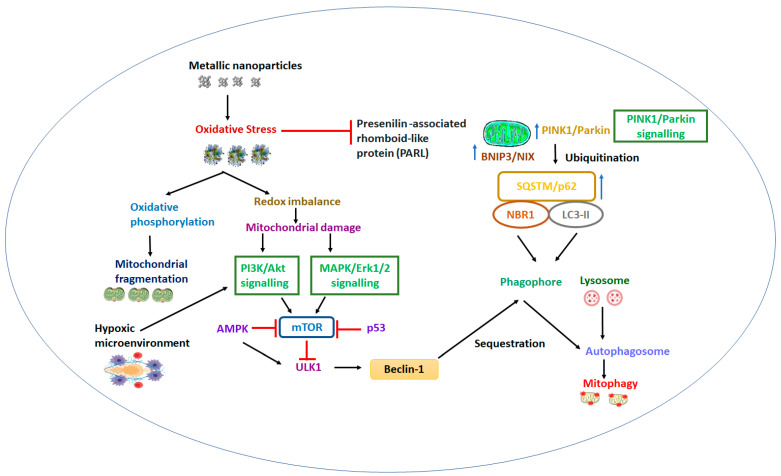
Schematic illustration on the mechanisms of metallic nanoparticles for targeting different pathway signals of mitochondria. PI3K: Phosphatidylinositol 3 Kinase; mTOR: Mammalian Target of Rapamycin; AMPK: Adenosine Monophosphate Activated Protein Kinase; Erk: Extracellular Signal-Regulated Kinase; Akt: Protein Kinase B; MAPK: Mitogen Activated Protein Kinase; ULK1: 112-kDa protein; BNIP3/NIX: Bcl-2 interacting protein 3; PINK1/Parkin-PTEN-induced kinase 1/autoinhibited E3-Ub ligase; SQSTM/P62: Sequestosome 1/p62 scaffold protein; NBR1: Ubiquitous Scaffold Protein; LC3-II: Ubiquitin-Binding Domain (Phosphatidylethanolamine conjugate).

Metal-based nanoparticles of sizes ranging between 5 and 260 nm were suitable for entry into the mitochondrial matrix. Wang et al. synthesized gold nanorods, in addition to discussing the comparative cellular internalization and intracellular trafficking through the mitochondria, lysosomes, cytoplasm, and endosomes of both normal and cancerous cells. A seed-mediated growth method was utilized to design cetyltrimethylammonium–ammonium bromide wrapped gold nanorods. The average particle size and zeta potential of developed nanorods were 18 ± 0.5 nm and +29.3 ± 0.7 mV, respectively. The prolonged retention and cationic surface charge of the nanorods reduced the mitochondrial membrane potential and generated reactive oxygen species in the mitochondria of lung cancerous cells (A549 cells), which caused apoptosis or cell death [51]. Figure 3 outlines the principal mechanism of metallic nanoparticles for targeting mitochondria.

However, the toxicity of metallic nanoparticles is reliant on biophysical characteristics, such as size, surface area, charge, and aggregation. These features affect biodistribution and internalization within organ systems, in addition to changing molecular interactions with receptors or macromolecules [52]. Published reports have suggested that there is a direct correlation between the particle size of nanoparticles and their efficiency to generate ROS in vital organs. For instance, silver nanoparticles with 10 nm size displayed superior biodistribution and caused more fatal effects in the liver and spleen cells, compared to the size range 40–100 nm [53]. As with the nanoparticle size, shape also affect biodistribution and clearance from the body. Long fibrous metallic nanoparticles exhibited more serious effects and were difficult to remove out from organ systems [54]. Similarly charged nanoparticles show greater accumulation in the target site compared to the noncharged ones. Peak et al. investigated the effects of particle size and surface charge of zinc oxide nanoparticles on pharmacokinetic parameters, including biodistribution and clearance upon administration of a single oral dose to rats. It was evident that negatively charged zinc oxide nanoparticles were extensively absorbed by the systemic circulation, compared to those that were positively charged. Zinc oxide particles of 20 nm were swiftly eliminated through the biliary and fecal routes [55].

### 5.1. Gold Nanoparticles (AuNPs)

In recent years, studies on AuNPs in both the healthcare sector and pharmaceutical research have boomed. Both bioaccumulation and cytotoxicity of AuNPs inside the eukaryotic cells are fascinating researchers. Lopez-Chaves et al. determined tissue dissemination, subcellular spotting, and toxic effects exhibited by gold nanoparticles (mean size 10–60 nm) on HepG2 and HT-29 cell lines. Inductively coupled plasma mass spectrometry was utilized to estimate bioaccumulation of gold inside the essential organs (i.e., liver, kidney, intestine, and spleen). However, performing transmission electron microscopy revealed their subcellular location in colon cells. These AuNPs were quite active for the overproduction of reactive oxygen species and free radicals, initiating lipid peroxidation and protein carbonylation, in addition to the depletion of DNA. The lowest sized AuNP exhibited more deleterious effects in the mitochondria and nucleus. Their pronged retention lessens mitochondrial potential and augments reactive oxygen species level, which mediates apoptosis, and is required for the designing of organelle-targeted nanoplatforms for chemotherapy [56]. Salnikov et al. explored calibrated AuNPs to sense the outer mitochondrial membrane that serves as the last barrier between the cytoplasm and mitochondria. The particle size was monitored, and the results suggested that, under normal (control) conditions, the outer mitochondrial membrane of the rats’ permeabilized ventricular cells was impermeable to 6 nm sized gold nanoparticles. That said, a 3 nm particle size was capable of entering the intermembrane space of mitochondria of isolated cardiac tissue 20 times more efficiently than the permeabilized ventricular cells. Further, voltage-dependent anion channel, 4,4′–di-isothiocyanatostilbene-2,2-disulfonic acid, and Konig polyanion tend to inhibit the entrance. Results gathered from green fluorescent protein concluded that the low availability of voltage-dependent anion channel (VDAC) within the outer mitochondrial membrane of cardiac mitochondria comprised a pore diameter >3 nm but <6 nm [57]. Gallud et al. studied responses of functionalized AuNPs on human monocytic cells. Both transcriptomics and proteomics concepts were included to demonstrate the cytotoxic effects of alkyl ammonium bromide, polyethylene glycol, and alkyl sodium carboxylate surface supplemented AuNPs. The results outlined a pronounced cytotoxic action and mitochondrial-dependent effects from the cationic ammonium terminated AuNPs, revealing an aggravated autophagy in RFP-GFP-LC3 receptor cells. However, no significant cell death was noticed in human leukemia monocytic cells (THP-1 cells) through PEGylated and carboxylated AuNPs [58]. Chen et al. developed a nanocomposite containing a gold nanostar and doxorubicin (anticancer drug). Hyaluronic acid is coencapsulated to protect the shell and enhance both the phototherapy and chemotherapy action. This nanosystem was modified, with proapoptotic peptide being added via sulfur bond. The nanocomposites were regular, with an average mean size of 40 nm. The decorated cationic peptide R8 and mitochondrial targeted triphenyl-phosphonium-KLA peptide did not disturb either the anatomy or the morphology of mitochondria. It was also observed that synthesized nanoplatform (i.e., AuNS-pep/DOX@HA) was successfully internalized into the cancerous cells via mediation of cell surface adhesion receptors (CD44). Further, coated hyaluronic acid was digested by the hyaluronidase enzyme, facilitating the release of doxorubicin in the subcellular site (mitochondria) after near-infrared light triggering photothermal therapy in vivo [59].

### 5.2. Iron Oxide Nanoparticles

To safely and effectively target mitochondria, a mitoNano approach encompassing different nanoparticles displays a potent role in the advanced management of cancer. This approach overcomes issues such as drug resistance and side effects. Classical multifunctional chemotherapeutic-embedded metallic nanoparticles are modified and functionalized to tailor their biocompatibility, as well as being potentiated with the effect of light. This photothermic or photodynamic property facilitates the selective delivery of metallic nanoplatforms into the mitochondrial cells [60].

Iron oxide nanoparticles are a widely explored nanoplatform, owing to their exclusive superparamagnetic properties. Despite being biocompatible, they have excellent ROS productive capacity, due to their ability to cause Haber–Weiss reactions. Produced highly reactive hydroperoxy radicals trigger oxidative stress and disrupt the DNA that mediate mitochondrial malfunction. Interestingly, iron oxide nanoparticles have the potential to behave like enzymes and catalyze reactions in presence of hydrogen peroxide and acidic solution, resulting in the production of highly toxic hydroxyl radicals that can be applied in cancer management [61]. Khan et al. synthesized iron oxide nanoparticles (size range 30–65 nm) using the sol–gel method to induce autophagy selectively in the A549 cancerous cells. Autophagy is a catabolic process that follows from the breakdown of carbohydrates, proteins, and lipids. It is exclusively related to the protein degradation system where cell protein contents and subcellular components are assimilated by lysosomal hydrolases, with the resultant production of ROS and mitochondrial abnormalities. In normal cells, autophagy maintains homeostasis via expelling out superfluous proteins and removing damaged or injured mitochondria, but it is highly important in the arena of chemotherapy research. Iron oxide nanoparticles have recently been acknowledged to induce autophagy by virtue of their cytotoxic action in mesenchymal stem cells, HeLa cells, A549, and fibroblasts of isolated lung tissue (IMR-90 cells) [62].

Zhang et al. investigated comparative autophagy effect using magnetic iron oxide and its PLGA-coated nanoparticles. All nanoparticles (i.e., iron oxide, PLGA-coated iron oxide, and PLGA nanoparticles) were successfully internalized via a cellular endocytosis process. In the same manner as magnetic iron oxide nanoparticles, comprehensively impaired lysosomes caused mitochondrial damage and cell organelle stress which mediated LC-3 positive autophagosomes, whereas PLGA-coated iron oxide nanoparticles exhibited marginal effects on these organelles (Golgi body and endoplasmic reticulum). Further, the cytotoxic study in mice revealed greater autophagosome accumulation of magnetic iron oxide nanoparticles in the kidney and spleen, compared to PLGA-coated iron oxide and bare PLGA nanoparticles [63]. Rivas-García et al. targeted mitochondrial DNA through synthesizing iron nanoparticles measuring 2–4 nm in size. The effect on both mitochondrial function and the process of autophagy were monitored in colorectal cell lines (i.e., HT-29). Iron nanoparticles were capable of entering subcellularly (mitochondria), which affected both respiratory and glycolytic activities. Autophagy caused by iron particles was analyzed through fluorescence signal determination. Two different dyes (such as Hoechst33342 and Cyto-ID^®^ Green dye) were employed, working as a subcellular permeation indicator and a green, fluorescent reagent in the treated cells (HT-29), respectively. Cells containing iron nanoparticles exhibited pre-eminently high autophagic vacuoles and intense fluorescence compared to the control. Further, iron nanoparticles were 62-fold more internalized in mitochondrial regions. Transmission electron microscopy revealed autolysosomes after 2 days (48 h) and concluded that exposure to small iron particles reduced number of MtDNA in colorectal tumor cells [64]. Ferroptosis therapy is concerned with cell death due to the deposition of iron-dependent lipid hydroperoxides. This new therapy has found application in the management of epithelial cell carcinoma. A nanodevice specific to the epithelial-to-mesenchymal transition has been designed, that worked on lipid peroxidase burst. The nanosystem was assembled on a mitochondrial membrane that had been magnetically sensitized. Sorafenib loaded in the self-assembly of supramagnetic iron oxide nanoparticles enhanced membrane permeability. The developed system released ferrous and ferric ions in the acidic environment of lysosomes that led to the bursting of the lipid known as hydroperoxidase. Approximately 18-fold higher accumulation was observed in the treated group of breast cancer cells. This ferroptosis therapy proved to be an alternate strategy for managing resilient epithelial cell carcinoma [65].

### 5.3. Silver Nanoparticles

Silver nanoparticles (AgNPs) have found extensive application in pharmaceutical sciences owing to their superb antibacterial and cytotoxic activities. AgNPs display exceptionally superior physicochemical properties, making them a highly fascinating and demanding metallic particle in the sectors of biomedical research and healthcare. Their pre-eminent antimicrobial activity is well established, while the development of a myriad of drug delivery systems is being explored. Nevertheless, the reported cytotoxic effects have elicited public concern with regard to their use in cosmetics and other household products. The literature contends that uptake and cell internalization of silver nanoparticles are via a clathrin-mediated process (a kind of endocytosis) within both lung fibroblasts and glioblastoma cells. Scanning electron microscopy revealed the uniform distribution of silver nanoparticles in the cell organelles. In nuclei, it causes a mitotic arrest that results in chromosome instability. Additionally, in mitochondria, AgNPs upset calcium transients through the activation of catabolic enzymes. In cancerous cells, silver nanoparticles inhibit cell proliferation by disturbing the signaling cascade pathway [66]. Recent research has focused on the wide application of silver nanoparticles to managing multidrug resistance in chemotherapy.

A study of the size-dependent cellular functions of silver nanoparticles (5 nm and 75 nm) was conducted by Gopisetty et al. in 2019, concluding that a particle size 75 nm had the potential to significantly inhibit the p-gp transporter efflux, disrupt mitochondria, create endoplasmic reticulum stress, and suitably manage drug-resistant breast cancer. The same effect was not observed in silver nanoparticles of 5 nm. However, both sizes of nanoparticles were quite sufficient to produce ROS and mitochondrial impairment [67]. Hou et al. have elaborated role of autophagy in the AgNP-induced cytotoxicity in the breast epithelium cells (HC11) of mice. HC11 cells released lactate dehydrogenase, which is predominantly associated with oxidative stress due to the augmented expression of hemoxygenase-1 and the liberation of both the reactive oxygen species (ROS) and nuclear factor erythroid 2–related factor 2 (Nirf-2). Further, mitochondrial membrane potential was found to be drastically reduced, whereas the level of parkin protein surged. Resultantly, the PTEN induced putative kinase (PINK 1) both activated and depolarized the mitochondria that lead to autophagy. Moreover, the administered silver nanoparticles influenced heterotrimeric AMP-activated protein kinase, which acts as sensor of cellular energy in mammalian adipose tissue. The protective autophagy in mitochondria lessened the expression of p-mTOR and p-Akt in HC11 mammary cells. The results suggested that silver nanoparticles can deteriorate mammary cells in clinical applications, which would impact lactation functions; it is hence suggested to be used with caution [68].

Another study on the effect of AgNPs on the mitochondrial redox mechanism was carried out in a panel of myriad lung cell lines, such as Calu-1, A549, NCI-H358, and BEAS-2B. AgNPs instigated the cell cycle, reducing proliferation of the cells of Calu-1, A549, and BEAS-2B. NCI-H358 cell lines were shown to be resistant against the action of AgNPs. Both the mitochondrial ROS and protein oxidation were enhanced upon being treated with AgNPs in a time- and dose-dependent manner [69]. Robust evidence supporting mammalian cells toxicity with the use of AgNPs has been reported in several literature reports and research. AgNPs are extremely associated with the generation of excessive ROS and a lowered level of ATP by upsetting cellular oxidant and antioxidant stages. Consequently, mitochondrial dynamics are disturbed, which involves mitochondrial fission, autophagy, apoptosis, and further cell death. The mitochondrial outer membrane proteins comprise fission proteins (i.e., mitofusin 1, mitofusin 2, and mitochondrial fission factor), whereas the inner membrane of mitochondria contains fusion protein (optic atrophy 1). Fission proteins mediate mitochondrial fission by employing GTPase dynamic related protein 1. Li et al. studied exposure of AgNPs in human nontumorigenic cells (HepG2 cells), which resulted in the alteration of mitochondrial morphology and biogenesis. AgNPs were treated with polyvinylpyrrolidone and had an average mean size of 23.44 nm, with a uniform shape. In Dulbecco’s Modified Eagle media, the particles exhibited improved size (51.58 nm) due to hydration. A cell viability study outlined a significant reduction in HepG2 cells in a time- and concentration-dependent manner, i.e., 20–640 μg/mL of silver nanoparticles for 24–48 h. The half inhibitory concentrations (IC50) of the designed silver nanoparticles in HepG2 cells after 24 h and 48 h were 1317.26 µg/mL and 301.6 µg/mL, respectively [70].

Though metallic nanoparticles are proven strategies for the deregulation of mitochondrial anatomy and functions, nanoparticles with antioxidant properties may induce mitophagy owing to their ability to alter both the signaling pathways (P13K/Akt/mTOR and Perkin pathway) and the levels of reactive oxygen species. Their optical, thermal, and physicochemical features have been explored, to combat various cancers and other critical ailments. Their enormous surface area and the presence of uncoordinated sites on their surface allows them to bind with therapeutics and bioactive agents, and thus perform catalytic action, facilitating precise delivery at the target site [71]. Their subcellular penetration efficacy (specifically towards mitochondria) makes metallic nanoparticles sensational in the biomedical research arena. The membrane potential difference and inhibition of mitochondrial respiration are major issues that can create apoptosis, mitophagy, and induce cell death in cancerous cells [72]. The mitochondrial dysfunction leads to cancer progression that involves several processes, including interrupted oxidative phosphorylation, reduced energy, reactive oxygen species accumulation, and inflammation in the cells. Therefore, mitochondria targeting is the emerging strategy to manage chemotherapy. Metallic nanoparticles, such as silver and zinc oxide, perform as an autophagy modulator and create a more stimulating signaling cascade in the tumor cells compared to normal cells [73].

**Figure 4:**
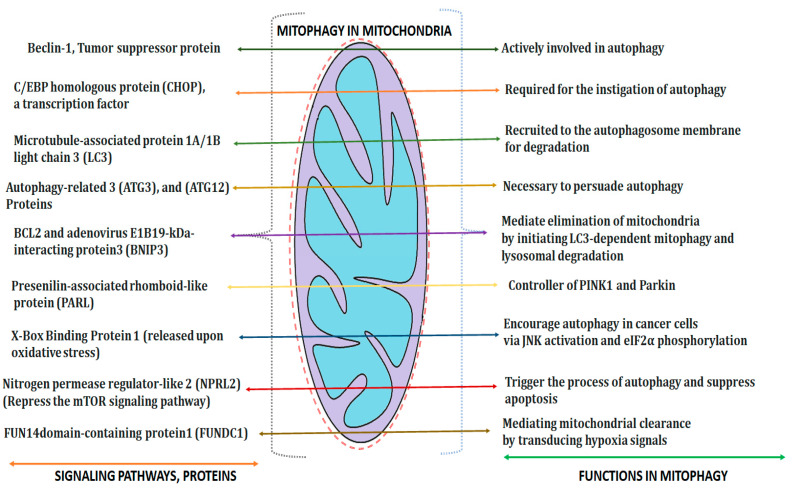
Various signaling pathways and their respective roles for ensuing mitophagy.

Skalska et al. investigated pathological alterations, such as cristolysis and swelling in mitochondria, that presented in rat brains after exposure to AgNPs at 0.2 mg/kg concentration. Further, an augmented expression of beclin-1 and MAP LC3-II (protein markers) were observed to induce autophagy [74]. Several mitophagy adaptors, such as a parkin, PINK1, bifunctional mitochondrial protein (BNIP3), autophagic indicator (P62/SQSTM1) and signaling pathways (nuclear factor (erythroid-derived 2)-like 2 (Nrf2)/PINK1/Parkin pathway), are impaired in cancer [75]. Piao et al. proposed the crucial role of silver nitrate nanoparticles in the suppression of glutathione level in the Chang liver cells of humans. Apart from ROS generation, both lipid membrane peroxidation and DNA fragmentation were also observed upon exposure to AgNPs. Thereafter, mitochondria dependent apoptosis (via the disturbance of mitochondrial membrane potential), the release of cytochrome C, the activation of caspases 9 and 3, and the variation on Bax/Bcl-2 expression were evident [76]. The potential application of AgNPs against human hepatoma (HepG2 cells) is also reported. Primarily, it induced mitochondrial swelling and enhanced intracellular level of ROS, which caused a collapse in membrane potential and mediated apoptosis via mitochondrial pathway. AgNPs concomitantly increased the autophagy marker proteins, such as LC3B and p62, that activate cytotoxicity in tumorous cells. Figure 4 explains the numerous signaling pathways and their functions involved in the process of mitophagy.

Breast cancer has garnered much interest among researchers. Although numerous therapies and treatments based on AgNPs are clinically approved, none of them completely cured the disease, nor proved to be a game changer. AgNPs are aggressively explored for their antitumor effect on the MCF-7 cell line. A study reported that AgNPs with a size of 9 nm successfully localized within the mitochondria and endoplasmic reticulum. This changed the mitochondrial dynamicity, depolarized membrane potential, generated ROS, and destabilized homeostasis. Resultantly, mitochondrial fission was initiated in breast cancerous cells. A study of their cytotoxicity revealed the apoptosis of MCF-7 via controlling the dynamicity of mitochondrial-ER [77]. Table 4 summarizes conjugated metallic nanoparticles and their mechanisms for targeting the mitochondria of cancer cells.

### 5.4. Titanium Dioxide (TiO_2_) Nanoparticles

TiO_2_ nanoparticles are extensively employed in different sectors, including food additives, cosmeceuticals, and environmental decontamination. However, the excessive and irrational usage of titanium dioxide creates serious health issues, including epigenetic changes in lung fibroblasts, hippocampal synaptic plasticity, and impaired placentation [97]. Nanoparticles of titanium dioxide were first explored in the year of 1990 for tissue engineering and drug delivery system applications. Recently, a lot of modifications have been brought TiO_2_ to lessen the risk of its tumorigenic property. In this series, mitochondrial dysfunction (due to excessive oxidative stress) is also determined with TiO_2_. The literature supports the understanding that imbalanced fission and fusion with the exposure of TiO_2_ results in both structural and functional disturbances in mitochondria [98].

Yu et al. demonstrated the toxicological outcomes of TiO_2_ nanoparticles (250 nm) in human bronchial epithelial cells that had been treated with a concentration range of 50–100 µg/mL for the period of 48 hrs. These nanoparticles trigger ROS generation and imbalance oxidative homeostasis inside the cells. The obtained results accurately described the destruction of mitochondrial-based endoplasmic reticulum membrane potential, as well as the disturbance of Ca^2+^ dynamics and the induction of endoplasmic reticulum stress inside the cell, mediating autophagy. This disturbed cellular homeostasis led to protein misleading, and eventually to the apoptosis or even the cell death of bronchial cells. Contrary to this, treatment of human bronchial epithelial cells with an ER stress inhibitor (Tauroursodeoxycholic acid) alleviated the toxic responses caused by TiO_2_ nanoparticles. This study recommends the performance of an exhaustive investigation on the harmful effects caused by TiO_2_ nanoparticles on the mitochondria, to ensure its safe and effective application [99].

Exposure of the skin to titanium dioxide is harmful in a daily routine and requires attention for the researchers when designing newer formulations. The various exposure pathways of TiO_2_ (including inhalation and ingestion) may create serious issues to human skin. Brassolatti et al. evaluated the cytotoxic action of functionalized TiO_2_ nanoparticles on human fibroblast (HDFn) and keratinocyte cells (HaCaT). The developed system contained a sodium carboxy ligand being added to the surface of TiO_2_ nanoparticles. An MTT assay was performed to investigate mitochondrial activity in both the cells, revealing that at elevated concentrations (>100 µg/mL) severe destruction of cell membrane occurred, due to generation of ROS within 72 h. The results suggested that a concentration below than 10 µg/mL was safe, and no cell alteration was reported [100]. To investigate the efficiency of TiO_2_ nanoparticles across brain cells, an exhaustive study was carried out by Huerta-García et al. in human and rat glial cells U373 and C6, respectively. Titanium dioxide nanoparticles disturb the redox signaling process and the peroxidation of lipids. High oxidation stress in both glial cells exhibited surged levels of oxidative enzymes, including glutathione peroxidase, superoxide dismutase, and catalase, which resulted in morphological changes, such as damage in mitochondria, and high membrane potential. The results revealed that TiO_2_ may enter into the brain cells via the olfactory bulb, where they are deposited in the hippocampus site, and hence exhibit cytotoxic action [101].

Zhao et al. investigated the alterations of mitochondrial dynamics due to exposure to TiO_2_ nanoparticles in the hippocampal neuronal cell line (HT22 cells). The results obtained through both the stimulated emission depletion and confocal microscopy demonstrated noticeable changes in mitochondrial length/density (submitochondrial structure) upon treatment with TiO_2_ nanoparticles. The nanoparticle causes a dynamic imbalance in the expression of optic atrophy 1 and dynamic related protein 1. Thereafter, TiO_2_ nanoparticles stimulate mitochondrial-based apoptosis via changing the signal pathways, including Bax, Bcl-2, and caspase 9 in the selected HT22 cells [102]. In the inner membrane of mitochondria, a special phospholipid called ‘cardiolipin’ is found that critically regulates the electron transport chain. TiO_2_ nanoparticles (10 nm) downregulate the cardiolipin profile and cause dysfunction in ATP production. Consistently, exposure to these nanoparticles significantly augmented dose-dependent ROS accumulation in mitochondria of bone-marrow-derived macrophages. Chen Q et al. quantified metabolic flux using a tracer (carbon 13 labelled glutamine) in a TCA cycle. The results described the dose-dependent decrement in the TCA metabolites, which was also supported by the reduced level of cardiolipin [103]. A thorough study on the molecular mechanisms and effect of TiO_2_ indicated that (around the tumor) microenvironmental influences are revealed, including metabolic changes, protein degradation, and gene mutation [104].

### 5.5. Zinc Oxide (ZnO) Nanoparticles

ZnO nanoparticles are widely employed in a diverse range of industrial sectors, including food additives, electronics, cosmetics, sunscreen products, and drug delivery approaches. These particles also possess anticancer and antimicrobial properties, owing to their efficient absorbance of ultraviolet and transparent visible light [105]. The wide applications of consumable products containing zinc oxide create alarming health issues. Sharma et al. demonstrated prominent adverse effects on liver, a primary site of metabolism in living beings. Both the genotoxic and apoptotic potential of zinc oxide was observed in HepG2 liver cells at a concentration of 14–20 µg/mL for 12 h. Significant decrement in cancer cell viability, due to induction of oxidative stress and DNA damage, was reported. Released reactive oxygen species lessened the mitochondria’s membrane potential via a surge in the Bax/Bcl2 signaling pathways. Further, the activation of p38, p53, and oxidative phosphorylation were also reported [106]. Nevertheless, the ion shedding property of ZnO nanoparticles categorized it as one of the most toxic and hazardous metal oxides compared to others (iron oxide, titanium dioxide, and aluminum oxide)**.** Clinical studies performed in different eukaryotes and prokaryotes revealed prominent cell toxicity. ZnO nanoparticles are efficient for targeting and transporting anticancerous therapeutics (such as methotrexate) in the vital organs, such as the liver, lungs, and heart. The growing evidence of systemic inflammation and cardiovascular toxicity was predicted in the literature, due to permeation potential of ZnO across biological barriers. Oral exposure of ZnO nanoparticles disrupts DNA and elevates levels of both creatine kinase and troponin in rat hearts, whereas inhalation and intrathecal instillation create fibrosis, inflammation, and necrosis in rat myocardium [107,108]. Liang et al. have designed ZnO nanoparticles and investigated the associated cytotoxicity in human aortic endothelial cells. A dose- and time-dependent intracellular oxidative stress and cell membrane leakage were achieved at the concentration of 8–50 µg/mL after a day, in human aortic endothelial cells. Cytotoxicity was observed with undissolved Zn^(2+)^ions, which induced apoptosis and necrosis after 24 h due to generation of ROS, which likewise impaired negative membrane potential, followed by the release of Cytochrome C, triggering caspase 3 and 9, and surging the ratio of Bax/Bcl-2 [109]. Exposure of ZnO against different cell lines, such as breast cancer and liver cancer MCF-2 and HepG2 cells, respectively, were investigated by Wahab et al. A nonprotonated chemical route was employed for the development of nanoparticles of size 13 ± 2 nm. The MTT assay revealed dose-dependent (2.5–100 µg/mL) cytotoxic activity and significant antiproliferation actions (95% and 96%; *p* < 0.05) against both HepG2 and MCF-7 cell lines. Fluorescence activated cell sorting (FACS) described a reduction in cell viability, whereas a real-time polymerase chain reaction demonstrated a substantial upregulation level of Bax, caspase 3, and p53, and a downregulation of Bcl-2, an antiapoptotic gene [110]. Bai et al. developed crystalline ZnO nanoparticles (20 nm) for the investigation of cytotoxicity and cell death (autophagy) in ovarian cancer. 

A series of cellular assay/tools were engaged to define the mechanism behind the mitochondrial damage and apoptosis. A dose-dependent damaged cell viability (due to the disturbance of mitochondria’s membrane potential and the generation of ROS or oxidative stress caused by the ZnO nanoparticles) were observed in treated ovarian cancerous cells. Damaged DNA strands were evident from the overexpression of γ-H_2_AX and Rad51 numbers. The Western blot analysis revealed the upregulation of p53, Bax, LC3, and caspase 9 with exposure of zinc oxide nanoparticles, which indicated the process of mitophagy and autophagy [111]. Zinc is also a common constituent of metal–organic framework (MOF) structures assembled from metal ions and polydentate ligands [112], and has recently been utilized in a new encapsulation technology that can deliver bioactive mitochondria inside cells. This novel technology is envisioned to be used for mitochondria, opening up a completely new avenue for the management of diverse mitochondrial diseases [113].

### 5.6. Selenium Nanoparticles

One of the rarer metalloids, selenium (allied of sulfur and tellurium) has also been explored for the cancer prevention by virtue of its ability to induce ROS-mediated apoptosis in malignant cells. Moreover, it causes an antioxidation process in healthy cells, in very low doses. These properties attract researchers to its use in targeting mitochondrial cells for the improvement of anticancerous efficiency. However, tissue toxicity is always a matter of concern when designing mitochondrial-targeted strategies with selenium nanoparticles [114].

Recently, the usage of selenium nanoparticles has been reported in the neurological abnormalities where neurons are destroyed by oxidative stress. The literature supports the understanding that functionalized and nonfunctionalized selenium nanoparticles proved a better alternative for managing oxidative stress [115,116]. Varlamova et al. have developed a nanocomplex (100 nm) using selenium nanoparticles and taxifolin (a naturally occurring antioxidant) for the regulation of mitochondrial calcium dynamics. Taxifolin is a recognized natural remedy for ischemic pathologies, owing to its potential neuroprotective effect. Its compromised pharmacokinetic parameters (poor solubility, low permeability across BBB, and toxicity) limit its frequent use in the design of targeted formulations. The authors developed different nanoparticles containing bare taxifolin (TAX), selenium (Se), as well as with their amalgamation (nanocomplexes of SE–TAX), before evaluating them for their possible efficiency with respect to the suppression of ROS generation in the astrocytes and neurons that had been exposed to hydrogen peroxide exogenously. Results confirmed the comparatively high efficiency of the prepared nanocomplexes SE–TAX in significantly reduced concentrations, by the virtue of selenium, which activated antioxidant enzymes and restrained the ROS production system. It was also revealed that the developed nanocomplex inhibited the rise of calcium (Ca^2+^) in cytosol, and that oxygen–glucose deprivation (OGD) provoked hyperexcitation in the neurological system. Hence, the system exhibited potential efficacy for the regulation of Ca^2+^ dynamics in the cell organelle, in addition to antiapoptotic effects and antioxidative properties [117,118].

Another vital application of selenium conjugated with Lentinan was reported by Lie et al. Lentinan, a kind of β-glucan, is isolated from *Lentinus edode* and enriched with variety of pharmacological activities, before being employed for the management of malignant ascites. Lentinan-functionalized selenium nanoparticles were synthesized; they were biocompatible and exhibited apoptosis-inducing effects for combatting cancerous ascites. The modi operandi behind necroptosis were observed as comprising the degradation of lysosomes and mitochondrial dysfunction via targeting (TRAF3)/mitofusin (MFN1) pathway, as well as in the high-grade serous ovarian adenocarcinoma cell line (OVCAR-3 cells) [119]. The overproduction of free radicals, damaged cells, and reduced immunity worsen the case of type II diabetes, and may lead to the incidence of cancers. The antioxidant selenium nanoparticles proved to be effective, after being combined with antidiabetic therapeutics, for the management of severe cases of diabetes. Yang et al. developed metformin–selenium nanoparticles with association of tween 80 to observe its synergistic effect on cancerous cells (MCF-7). An upregulation of p38, p-ATM, and p-ATR, as well as a downregulation of cyclin-dependent kinase were noted, which caused cell cycle arrest. These processes controlled mitochondrial membrane potential [120].

## 6. Conclusions and Prospects

Subcellular mitochondria perform many of the most vital applications, owing to their being the powerhouse of the cell. Different potential uses, such as directing diverse signaling pathways, ATP generation, regulating mitochondrial protein synthesis, and their control of cellular events (cell proliferation, apoptosis, and cell death) in eukaryotic cells are particularly governed by mitochondria. These roles make mitochondrial participation for the management of different diseases (including diabetes, cardiac and neurological disorders, as well as tumor progression) crucial. Nanoengineered systems can offer vital applications for the management of mitochondrial-based disorders, owing to their enhanced surface area and controllable size, which can be tuned so as to easily permeate and deliver therapeutics inside the mitochondrial architecture. Stable and biocompatible metal-oxide-based nanoparticles have found promising applications in the mitigation of various ailments, including destructive cancers. They can serve as chemotherapeutics in the same manner as photodynamic therapy (PDT), sonodynamic therapy (SDT), radiotherapy (RDT), and cancer detection biomarkers. Metallic nanoparticles particularly overexpress exogenous/endogenous reactive oxygen species, and can trigger intrinsic cell necrosis via mitophagy, as well as autophagy via other exclusive mechanisms. For instance, iron nanoparticles can cause a sharp rise in temperature inside the mitochondria in cancerous environments, trigger apoptosis, and control cancer progression, whereas zinc nanoparticles raise creatin kinase and troponin levels, and can thus damage mitochondrial DNA. Similarly, silver nanoparticles upset calcium transience through activating catabolic enzymes inside mitochondria. Multifunctional functionalized metallic nanoplatforms can more precisely and selectively target the cancerous cells, compared to nonmodified nanoparticles, and can therefore eliminate the chance of poor lysosome escape and rapid clearance. Hence, metallic nanoparticles are expected to constitute a powerful weapon for the management of a myriad of ailments and disorders. However, this approach still faces different health-related issues in clinical trials, owing to their biocompatibility and potential toxicity issues towards healthy tissues. Attention is to be paid during synthesis (preparation), animal studies, and safety assessments of nanoparticles since their oxidation state may influence the activity on mitochondria. Hence, thorough information on the modus operandi, in vivo metabolism, pharmacokinetic parameters, and toxicological aspects should be gathered in order to design safe and high-potential metallic nanoparticles for the targeting of subcellular mitochondria.

## Figures and Tables

**Table 1:** Mitochondrial abnormalities and their management in different human diseases.

Disease	Mitochondrial Abnormalities	Management	Ref.
Cardiovascular diseases	Impaired mitochondrial electron transport chain due to elevated LDL, greater ROS production disturbed cardiac tension, altered cytosolic Ca^2+^ flux, ischemia-reperfusion injury, and other diseases (such as diabetes mellitus)	Control fatty acids and cholesterol level. Regulate enzymes i.e., mitochondrial creatin phosphate, creatin kinase, and ATP synthase. Modulate Ca^2+^ concentration in myocardium.	[8]
Diabetes	MtDNA mutation Delayed Electron transport chain Increased beta oxidation and lipid accumulation Higher ROS overproduction Disturbed insulin signal pathway Increased intracellular glucose content, and constrained metabolism of glucose	Prevention of ROS and lipid peroxidation. Reverse MtDNA change. Enhance glucose metabolism by inhibiting acetyl-CoA in mitochondria.	[9]
Kidney	Mitochondrial DNA mutation (complex I–V) Hypomagnesemia Hypokalemia Hypoparathyroidasim Uremic toxins Kidney diseases	Manage Erythropoetin signaling for normal mitochondrial biogenesis and metabolism. Hypoxia-inducible factor prolyl hydroxylase (HIF-PH) inhibitors, Nrf2-activating triterpenoid, sodium–glucose transporter 2 (SGLT2) inhibitors, and control of carnitine level	[10]
Alzheimer’s disease	Aggregated Aβ peptide bond with a component that controls mitochondrial permeability ‘cyclophilin D’ Resultant reduction in membrane potential due to opening of pores. Free energy and ROS generated by beta amyloid peptide. Leads to MtDNA mutation and neuronal toxicity.	Inhibition ofAβ peptide clusters binding with cyclophilin D. Restoration of MtDNA and enzyme replacement Inhibit cytochrome c and caspase activity Decrease mitochondrial fission	[11]
Cancer	Enhanced mitochondrial complex I activity. Mutations in oncogenes Expression of oncoproteins (IDH 1 and 2, SDH and FH) Apoptosis induced factors released. Defective oxidative phosphorylation Hypoxic milieu proliferate condition	Isocitrate dehydrogenase (IDH) 1 and 2 inhibitors Oxidative phosphorylation suppression Antioxidants Mitochondrial complex I and V inhibitors Lactate dehydrogenase (LDH) inhibitors	[12]

**Table 2:** Therapeutic agents explored for the management of impaired mitochondria.

Therapeutics	Approach	Model	Outcome	Ref.
**Upsurging ATP levels**				
Inosine and Febuxostat	To increase ATP level and hypoxanthine in peripheral blood	Patients with homoplasmic and heteroplasmic mutations	After oral administration, brain natriuretic peptide (specific marker for heart failure) was reduced up to 31%. Moreover, 3.1-fold insulinogenic index was improved, suggesting a promising action of the given treatment.	[13]
**Stimulating mitochondrial biogenesis**				
Bezafibrate and AMPK agonist 5-aminoimidazole-4-carboxamide ribonucleotide	To initiate biogenesis and activate AMP protein kinase/PGC-1α-dependent pathway	Double recombinant mice overexpressing PGC-1α in skeletal muscles	Stimulation of PPAR/AMPK/PGC-1 alpha increased mitochondrial biogenesis, which monitors the homeostatic pathway and motor improvement in Sco2(KO/KI) animal model	[14]
5-Aminoimidazole-4-carboxamide ribotide (AICAR)	ATP content and mitochondrial growth were aimed without disturbing membrane potential	CI deficient fibroblasts, such as NDUFS2 and C20ORF7	Fluorescence microscopy detailed the activation of AMP protein kinase with AICAR	[15]
Nicotinamide riboside (NAD+ precursor)	Effect of nicotinamide in pharmacokinetic parameters and NAD+ level in blood for the treatment of genetic or acquired mitochondrial abnormalities	Impaired mitochondrial murine model	Orally administered nicotinamide riboside was well tolerated, and an increased mean steady state concentration (Css, *p* = 0.03) two times greater than the baseline NAD+ concentration in blood was observed, whereas average circulating level of NAD+ at day 1 was 27 ± 6 µM.	[16]
**Modulation of the Nitric acid or cGMP/PKG pathway**				
L-arginine (Nitric acid precursor)	To reduce capacity for nitric acid-based vasodilation	MELAS (mitochondrial myopathy, encephalopathy, lactic acidosis, and stroke-like episodes) patients	Obtained data suggested that prepared L-Arg infusion was most effective when administered within 4 hrs of the onset of brain disorder symptoms in the acute phase of MELAS sufferers	[17]
Neural progenitor cells (NPC)	To preserve parental mtDNA and show metabolic shift toward oxidative phosphorylation	Homoplasmic mutation in the mitochondrial gene (MT-ATP6)	Human induced pluripotent stem cells originated NPC, providing a potential tool for mtDNA targeted drug screening to avoid nervous system disorder.	[18]
**Antioxidant therapy**				
N-acetyl cysteine	Supplementation with N-acetyl cysteine to modify the mitochondrial respiratory chain function	MELAS patients containing common mutation, i.e., m.3243A>G, m.8344A>G	Supplementation with N-acetyl cysteine improved 2-thiomodificationof tRNA, thus regulating protein synthesis.	[19]
Cysteamine bitartrate	Enhancement of glutathione biosynthesis for the reduction of oxidative stress associated with mitochondrial diseases	Zebrafish model and *Caenorhabditis* elegans model carrying Complex I defect	Cysteamine bitartrate improved mitochondrial membrane potential in nephropathic cystinosis and cured multiple RC complex diseases in FBXL4 human at 10 to 100 μm concentrations.	[20]
**Inclusion of Redox-Active Molecules**				
Tc99m-HMPAO	To explore Tc99m-HMPAO for determination of glutathione/protein thiol levels in cerebral blood flow	Pediatric mitochondrial diseased patients	The patients showed improvement in Newcastle score (n = 5, *p* = 0.028), confirming the potential of Tc99m-HMPAO as a bioimaging marker for the oxidative state of brain.	[21]
EPI-743	Management of cellular oxidative stress in mitochondrial respiratory chain disease	5-month-old girl suffering from Leigh syndrome	EPI-743 (a potent stress protectant) exhibited improvement in mitochondrial associated issues, i.e., improvement in eye, motor, and bowel movements	[22]
**Monitoring of mitochondrial dynamics**				
Cytotoxic Necrotizing Factor-1	To control mitochondrial impairment and cell damage	Patient sufferer from m.8344A>G gene	CNF-1 triggered energetic content of mitochondria via activation of actin cycloskeleton in Myoclinic epilepsy with ragged red fibers (MERRF) and increased mitochondrial marker tomo20	[23]
**Modulating mitochondrial autophagy**				
Rapamycin	Inhibition of mTOR in mitochondrial defect in Leigh syndrome	Ndufs4 wild type mouse	Rapamycin slowed down neurological symptoms and minimized neuroinflammation in brain lesions. It also slowed the formation of glycolytic intermediates.	[24]
NADH dehydrogenase Ndi1	To replace mitochondrial complex 1 for reoxidation of NADH intramitochondrially.	Transgenic strains of Drosophila	Overexpression of NDI1 relieves aging and manages production of ROS. Deposition of these oxidative damaged markers is declined in aged flies.	[25]

**Table 3:** Nanoengineered systems for mitigation of impaired mitochondrial diseases.

Nanodrug Delivery System	Purpose	Model	Outcomes	Relevance	Ref.
Topotecan loaded liposome	Mitochondrial targeted system to overcome resistant related metastases	Multi drug Resistant MCF-7/ADR cell xenografts	Mitochondrial targeted liposomes were 64.84 nm with −0.52 ± 0.08 mV membrane potential. Encapsulation efficiency was ≥95%. They were stable in physiological blood system and exhibited minimal leakage. The system led to release cytochrome C, stimulated caspase 9 and 3, and exhibited superior inhibitory action on the resistant B16 melanoma metastatic mice.	Topotecan localized in mitochondria that exhibited potent inhibitory action on the on the resistant B16 metastatic melanoma.	[44]
Paclitaxel loaded triphenylphosphine nanomicelles	Inhibition of antiapoptotic Bcl-2	Drug-resistant breast cancer-bearing mouse model with lung metastasis (A549/ADRcells)	The nanomicelles were small (142 ± 8.35 nm) with PDI 0.235 and negative zeta potential (−24.65 ). This system significantly hampered A549/ADR cells and deposited over mitochondria surface. Inhibition of Bcl-2 led to release cytochrome C and triggered caspase 3 and 9, mediating mitochondrial outer membrane permeabilization.	Positively charged nanomicelles adhered inside mitochondria and exhibited apparent multidrug resistant tumor targeting efficacy	[45]
Curcumin loaded polymeric and lipid nanosuspensions	To neutralize generation of reactive oxygen species due to disturbance of signal proteins in mitochondria.	Olfactory ensheathing cells	Uniform and spherical polymeric and lipid nanosuspensions were of mean sizes 338 nm and 127 nm, respectively, with negatively charged zeta potential. The nanosuspensions were quite stable for more than 135 days. Improved cell viability suggested potential incorporation of curcumin in nanosuspension against hypoxic cells of Olfactory ensheathing cells at the concentration of 5 µM.	Potential intranasal polymeric and lipid nanosuspention was advised for neuroprotective action due to antioxidant property of curcumin.	[46]
Hydroxyl-terminated polyamidoamine (PAMAM)-*N*-acetyl cysteine dendrimers	Mitochondrial targeted delivery in oxidative stress-induced glial cell	Rabbit traumatic brain injury (TBI) model	Significantly high localization of drug in mitochondria than nonmodified dendrimer due to potential for attenuation of oxidative stress. Systemic administration in TBI model of rabbit exhibited capability to penetrate BBB and target glial cells due to localization in the white matter of the injured hemisphere.	Colocalization of dendrimer in mitochondria of glial cells in traumatic brain injury	[47]
Osthole nanoemulsion (OST-NE)	Regulation of apoptosis pathway and mitochondrial oxidative stress.	Alzheimer’s disease model mice	Intranasal delivery of osthole nanoemulsion (mean particle size 2.33 nm) enhanced bioavailability, regulated cholinergic system, maintained mitochondrial potential, and inhibited apoptosis and oxidative stress.	OST-NE lessened upstream modulator Bax, that depolarized mitochondrial membrane potentail and exhibited antiapoptotic effect or neuroprotective action in Alzheimer disease	[48]
*Momordica charantia* silver nanoparticles	Uphold mitochondria biogenesis and enhanced the expression of PPARϒ, an energy metabolism coordinator	Pancrease of diabetic rats	Silver nanoparticles possessed irregular/uneven surface and diameter. Contained *Momordica charantia* enhanced glucose sensitivity in the dibetic rat model at a lower dose of 50 mg/kg by slowing down JAK/STAT and AKT/PI3K pathways in mitochondria.	Developed nanoparticles promoted glucose uptake and insulin secretion via improving mitochondrial biogenesis in pancreas of diabetic rats.	[49]
Dequalinium embedded DQAsomes	Dicationic amphiphilic was investigated for affinity towards binding with mitochondria DNA.	Plasmid DNA firefly luciferase	Developed DQAsomes created a liposome-like aggregate system in aqueous media that were able to bind with DNA and had efficiency to transfect cells compared to Lipofectin™ reagent.	DQAsomes selectively accumulated in mitochondria of cancerous cells, and suggeted potential use as a nonviral transfection vector in gene delivery system.	[50]

**Table 4:** Functionalized metallic nanoparticles explored for the management of cancer via targeting mitochondria.

Metallic Nanoparticles	Conjugated with	Mechanism and Model	Outcomes	Ref.
**Functionalized gold nanoparticles**				
Fluorescent gold nanoprobe	Fluorophore cyanine 5	Mitophagy, breast cancer cell line (MDA-MB-231)	Oxidation and polarization in the mitochondrial membrane, used for tracking lysosomes to image mitophagy	[78]
Gold nanoparticles	Tumor necrosis factor-related apoptosis-inducing ligand	Autophagy, Excessive Drp1-dependent mitochondrial fragmentation, and dysfunction in tumor cells	Promoted apoptosis in nonsmall cell lung cancer (NSCLC) cells	[79]
Epigallocatechin gallate capped gold nanoparticles	Poly-D-lysine-PEG functionalized with mitochondria targeting cation (triphenylphosphonium)	Mitochondrial Caspasedependent apoptosis in HeLa cell line. The laminin receptor dependent uptake of nanoparticles	Preferentially localized in mitochondrial and enhanced delivery of paclitaxel to the Cervical carcinoma cells	[80]
Epigallocatechin gallate-capped gold nanoparticles	Cationic triphenylphosphonium functionalized	Predominant mitochondrial depolarization, Activation of caspases 3 and 7, and G0/G1 phase arrest of the cell cycle. Cellular uptake and mitochondrial localization in human Caco-2, MCF-7, and HeLa cancers cell lines	Mitochondrial targeted delivery botulinic acid gold nanoparticles with lesser IC_50_ values (3.12–13.2 µM) compared to the bare one (9.74–36.31 µM). Mitochondrial apoptosis in cancer cells.	[81]
**Functionalized iron oxide nanoparticles**				
Iron oxide nanoparticle (fluorescent)	Coumarin	In HeLa cells, intracellular temperature was increased by 2.1 °C in five minutes on laser irradiation of 740 nm that targeted mitochondrial cells.	The system particularly released coumarin-derived iron oxide NPs (fluorescent) to the mitochondria of HeLa cells. Further, elevated temperature and more cytotoxicity mediated photothermal therapy and lead to better targeting in cancerous cells.	[82]
Iron oxide nanoparticles	TAT, a cell penetrating peptide	Encouraged leakage of electrons from the mitochondrial electron transport chain that led to amplified production of reactive hydroxyl radicals and permeabilized lysosomal membrane of cancerous A549 cells.	TAT modified iron oxide nanoparticles along with radiation therapy synergized effects of chemotherapy	[83]
Iron oxide	Carbonyl monoxide complex	Generation of carbon monoxide from complex initiated mitophagy/autophagy	Developed iron pentacarbonyl system specifically triggered under near-infrared irradiation in the tumor environment. Carbon monoxide and iron oxide damaged mitochondria and synergized effect of cancer cells.	[84]
**Functionalized silver nanoparticles**				
Silver nanoparticles	Glucose	Castrate-resistant prostate cancers cell line was selected, due to its capacity for high glucose consumption.	Developed system was stable, spherical, and had average size 61 nm. It produced ROS, depolarized mitochondrial membrane potential, and led to cancerous cell apoptosis.	[85]
Silver nanoparticles	Polydopamine-coated paclitaxel for targeting tumor peptide NR1	Augmented Bax-to-Bcl-2 ratio and the stimulation of proapoptotic P53 and caspase 3 pathways resulted in intense ROS release and a break down of double-stranded DNA.	NR1 decorated paclitaxel-silver nanoparticles displayed pH responsive drug release and superior apoptosis via mitochondrial membrane lysis and nucleus damage.	[86]
Silver nanoparticles	Tamoxifen	The conjugate stimulated cell apoptosis via mediating Bax/Bcl2 and caspase–cascade signal pathways in mitochondria.	Tamoxifen conjugated silver nanoparticles (440 nm) were amorphous, disrupted the mitochondrial membrane, and induced apoptosis signal in human breast cancer cells.	[87]
**Titanium dioxide nanoparticles**				
Mesoporous titanium dioxide	Nitric acid decorated L-arginine and mitochondrial targeting ligand (triphenyl phosphonium)	Conjugated nitric oxide inhibited cell respiration and released reactive nitric species targeted mitochondrial sites of cancerous cells (MCF-7).	Developed system efficiently accumulated inside the mitochondria and generated Nitric oxide gas and ROS. A synergized effect of sonodynamic therapy on the management of breast cancer achieved.	[88]
Titanium dioxide	Erlotinib and Vorinostat	Different cancerous cells, including MDA-MB-231, MCF-7 and cancerous amniotic cells. Depicted G2/M phase cell arrest.	Designed nanoconjugate exhibited induction of apoptosis due to deposition of p53. Increased ROS and disrupted mitochondrial DNA were exhibited in treated lymphocytes.	[89]
Titanium dioxide	Quercetin	The level of mitochondrial complex I–V dropped with ATP depletion in rodent model. in rodents. Enhanced levels of protein carbonyl and lipid peroxidation at 10 and 50 μg/mL concentration.	The conjugate acted as potential antioxidant and caused mitochondrial dysfunction and oxidative stress in liver mitochondria.	[90]
**Functionalized platinum nanoparticles**				
Platinum nanoparticles	Peptide coated TPP-Pt	Deeper tissue penetration with lesser photon scattering at 1.0 W cm^−2^ permissible exposure.	Developed conjugate was monodispersed, stable, and exhibited precise NIR II (1000–1350 nm) phototherapy in a low amount to a thermally susceptible mitochondrion. Photothermal therapy for the management of cancer	[91]
Pt-Ceria-8-atto nanoparticles (8 nm)	Conjugated with triphenyl phosphonium and Atto 647 N fluorescent dye	HMEC1 cell line. Conjugated lipophilic cationic molecule (triphenyl phosphonium) and dye (Atto 647 N) both attracted and accumulated on the mitochondrial negative membrane potential surface.	Selectively accumulated in the mitochondria. Nanoparticles larger than 150 nm (PDI < 0.1) were internalized via conventional process of endocytosis.	[92]
Aggregation-induced emission-based platinum IV	Triphenyl phosphonium	Augmented reactive oxygen species and lessened mitochondrial membrane potential. Cell cycle arrest in the S-phase and DNA disruption.	Potentially active for the management of solid tumor spheroids.	[93]
**Functionalized zinc oxide nanoparticles**				
Quercetin-decorated zinc oxide nanoparticles	Phenylboronic acid	pH responsive release pattern was observed using 40 nm sized nanoparticles (−10.2 mV zeta potential) in MCF-7 cells	The nanoconjugate induced enhanced oxidative stress, damaged mitochondria, and induced apoptosis in breast cancer cells.	[94]
PEG coated zinc oxide	Doxorubicin	Nanocomposite induced cell injury due to generation of ROS under UV irradiation in HeLa cell lines.	Photocleavage of DNA and photodynamic therapy in cancer management.	[95]
Zinc oxide nanoparticles with artesunate and glucose oxidase	Hyaluronic acid	Generated hydrogen peroxide caused oxidative damage and accelerated glucose oxidase in cancerous cells	Nanocomposite (163 nm) exhibited intrinsic affinity with CD44 receptors and produced cancer starvation	[96]

## Data Availability

Not applicable.
